# ecDNA within tumors: a new mechanism that drives tumor heterogeneity and drug resistance

**DOI:** 10.1038/s41392-020-00403-4

**Published:** 2020-11-24

**Authors:** Xixi Zeng, Maoping Wan, Jianmin Wu

**Affiliations:** Institute of Genomic Medicine, Wenzhou Medical University, Wenzhou, 325035 Zhejiang P.R. China

**Keywords:** Cancer, Translational research

**Fig. 1:**
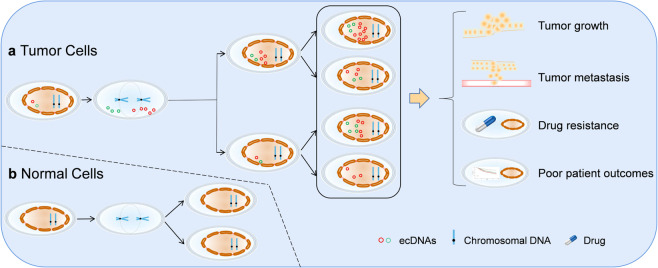
Schematic summary of roles of amplified ecDNAs in cancer. EcDNA-based oncogene amplification promotes intratumoral genetic heterogeneity and is associated with aggressive cancer behavior, including tumor growth, metastasis, and drug resistance, resulting in poor outcomes for patients across multiple cancers

A recent study by Hoon Kim et al. published in *Nature Genetics* demonstrated that extrachromosomal DNA (ecDNA)-based oncogene amplification frequently occurs in most cancer types and that it is different from chromosomal amplification.^1^ The authors further showed that ecDNA in multiple cancer types leads to poor outcomes in patients; thus, ecDNA is a novel potential diagnostic or therapeutic target for tumor treatment (Fig. 1).

Tumor cells adopt various unique biological mechanisms to drive their aggressive behavior and ensure survival. Early studies have documented that oncogenes amplified on extrachromosomal DNA (ecDNA) show high expression levels of mRNA transcripts.^2^ The extrachromosomal gene was first described by Yasuo Hoota and Alix Bassel in 1965.^3^ It was later named ecDNA, which is a class of circular DNA ranging from tens to millions of base pairs in size.^4^ Pan-cancer analyses revealed that oncogenes encoded on ecDNA were among the most highly expressed genes in the transcriptome of the tumors,^5^ suggesting that ecDNA may play pivotal roles in tumor progression.

Previously, Mischel et al. found that there were many cyclic ecDNAs in human tumor cells that played a key role in the rapid evolution of tumors and in their defense against threats such as chemotherapy, radiation, and other therapies.^2^ Furthermore, researchers have demonstrated that (1) ecDNA is circular, (2) ecDNA drives the expression of massive oncogenes, (3) ecDNA contains highly accessible chromatin, and (4) ecDNA has a significantly greater number of ultra-long-range interactions with active chromatin.^5^ Although ecDNA-based amplification has been shown to promote intratumoral genetic heterogeneity and accelerated tumor evolution, its frequency and clinical impact are still unclear.

In order to perform a global survey on the frequency of ecDNA-based oncogene amplification, the authors performed ecDNA prediction from whole-genome sequencing (WGS) data using AmpliconArchitect based on three characteristic properties of ecDNA: its circular nature, its highly amplified characteristic, and its lack of a centromere. After analyzing WGS datasets from 3212 tumor samples and 1810 non-neoplastic ones, the authors found that, among all tumor samples, approximately 14.3% carried one or more circular amplicons, which shows that ecDNA-based amplification is a common event in cancer. However, almost no circular amplifications were detected in matched whole-blood or normal tissue samples.^1^ Earlier, Paul S. Mischel et al. developed the ECdetect tool to conduct unbiased integrated ecDNA analyses of WGS datasets from 17 cancer types and showed that ecDNA was detected in nearly half of the samples.^2^ In this recent study, ecDNA-based circular amplicons were found in 25 of 29 cancer types analyzed; in particular, aggressive histological cancers harbored a high frequency of amplicons.^1^ All these results demonstrate that ecDNA amplification can be defined as a feature of multiple cancer subtypes.

Next, the authors found that the chromosomal distribution of the 579 circular amplicons was highly nonrandom. Then, they analyzed the association between 24 most recurrent amplified oncogenes and circular amplicons, and found that 38% of these genes were most frequently present on ecDNA amplicons.^1^ Furthermore, the authors found that ecDNA was formed through a random process, and most circular amplicon breakpoints showed no or minimal sequence homology, implicating nonhomologous end-joining (NHEJ) in ecDNA-associated breakpoint repair. Subsequently, the authors revealed that ecDNA formation can result from chromothripsis, which is associated with NHEJ. In order to examine the transcriptional consequences of circular ecDNA amplification, the authors investigated the correlation between DNA copy number (CN) and oncogene expression level. They found that ecDNA amplifications resulted in higher levels of oncogene transcription compared to CN-matched linear DNA.^1^ This is consistent with recent findings that oncogenes amplified on ecDNA have markedly increased numbers of transcripts in cancer cell lines and clinical tumor samples.^5^ To compare the epigenetic mechanisms governing gene expression between circular amplifications and noncircular regions, ATAC-sequencing profiles from 36 samples were analyzed. The authors revealed that enhanced chromatin accessibility played key roles in the dysregulation of ecDNA oncogenes and that ecDNA amplifications more frequently resulted in transcript fusions.

Furthermore, the authors used the lymph node status and gene expression signatures (increased tumor cell proliferation and reduced immune cell infiltration) to investigate the association between ecDNA and aggressive biological features. Their results revealed that ecDNA amplification groups showed more spread to lymph nodes at initial diagnosis, higher cellular proliferation scores, and lower immune infiltration scores. Most importantly, the authors found that the 5-year survival rate was significantly lower in patients whose tumors harbored ecDNA amplification, demonstrating that the presence of ecDNA was associated with aggressive cancer behavior. To illustrate how survival is related to disease subtypes, the authors used a multivariate Cox proportional-hazards model to test survival after controlling for disease subtype and found that circular amplification resulted in significantly higher hazard ratios.^1^ Together, these data imply that ecDNA amplifications may provide tumors with tolerance to clinical treatment and aid them in escaping from barriers to evolution.

In summary, this study revealed that circular ecDNA plays a key role in the development and progression of cancer by not only facilitating enhanced chromatin accessibility and the transcription of oncogenes but also adversely affecting the survival and outcomes of cancer patients.^1^ Oncogenes encoded on ecDNA untether themselves from their chromosomal constraints, which endows tumors with the ability to rapidly change their genomes in response to changing environments, thereby accelerating tumor evolution and contributing to therapeutic resistance. Because ecDNAs widely exist in different types of tumors, deep dives into the basic structure and function of ecDNAs will aid in understanding the mechanism underlying their biogenesis, replication, and trafficking. More importantly, it is promising to find new diagnostic makers depending on the presence of ecDNAs in tumors, as well as to develop innovative therapeutic strategies that can target ecDNA to interfere with their ability to drive tumor growth, drug resistance, and recurrence.
